# The clinical impact of *IKZF1* mutation in acute myeloid leukemia

**DOI:** 10.1186/s40164-023-00398-y

**Published:** 2023-03-30

**Authors:** Xiang Zhang, Aijie Huang, Lixia Liu, Jiayue Qin, Chengcheng Wang, Min Yang, Yinjun Lou, Lei Wang, Xiong Ni, Xiaoxia Hu, Gusheng Tang, Mengmeng Zhang, Shanbo Cao, Liping Mao, Jiejin Qian, Weilai Xu, Juying Wei, Gaixiang Xu, Haitao Meng, Wenyuan Mai, Chunmei Yang, Honghu Zhu, Hongyan Tong, Jianmin Yang, Wenjuan Yu, Jianmin Wang, Jie Jin

**Affiliations:** 1 Department of Hematology, The First Affiliated Hospital, Zhejiang University School of Medicine, #79 Qingchun Road, Hangzhou, 310003 Zhejiang People’s Republic of China; 2 Department of Hematology, Institute of Hematology, Changhai Hospital, #168 Changhai Road, Shanghai, 200433 People’s Republic of China; 3 Acornmed Biotechnology Co. Ltd., Tianjin, 301700 People’s Republic of China

**Keywords:** Acute myeloid leukemia, *IKZF1* mutation, Clinical impact

## Abstract

Genetic heterogeneity poses a great challenge to the understanding and management of acute myeloid leukemia (AML). Knowledge of the *IKZF1* mutation in AML specifically is extremely limited. In a previous work, we described the distribution pattern of *IKZF1* mutation in AML, but its clinical impact has remained undefined due to the limited number of cases. Herein, we attempt to answer this question in one relatively large cohort covering 522 newly diagnosed AML patients. A total of 26 *IKZF1* mutations were found in 20 AML patients (20/522, 3.83%). This condition has a young median age of onset of morbidity (P = 0.032). The baseline characteristics of *IKZF1*-mutated and wild-type patients were comparable. *IKZF1* mutation showed significant co-occurrences with *CEBPA* (P < 0.001), *SF3B1* (P < 0.001), and *CSF3R* (P = 0.005) mutations, and it was mutually exclusive with *NPM1* mutation (P = 0.033). Although *IKZF1*-mutated AML was more preferably classified into the intermediate-risk group (P = 0.004), it showed one inferior complete remission rate (P = 0.032). AML with high burden of *IKZF1* mutation (variant allele frequency > 0.20) showed relatively short overall survival period (P = 0.012), and it was an independent factor for the increased risk of death (hazard ratio, 6.101; 95% CI 2.278–16.335; P = 0.0003). In subgroup analysis, our results showed that *IKZF1* mutation conferred poor therapeutic response and prognosis for *SF3B1*-mutated AML (P = 0.0017). We believe this work improves our knowledge of *IKZF1* mutation.

**Supplementary Information:**

The online version contains supplementary material available at 10.1186/s40164-023-00398-y.

*IKZF1* mutation is one rare but recurrent alteration in AML. In a previous work, we described its distribution pattern in AML [1], but the clinical impact of *IKZF1* mutation on AML remains undefined. We here address this issue in a cohort of 522 newly diagnosed AML patients (Additional file 1: Fig S1, Patients and methods in supplementary information).

Recurrent *IKZF1* mutation, including 12 missense mutations, 4 nonsense mutations, and 10 frame-shift mutations, was found in 20 patients (3.83%). Missense mutation preferred to localize at the exon 5 (91.67%), which mainly influences the DNA binding of IKZF1. A total of 35.7% of nonsense and frame-shift mutations were found to disrupt the DNA-binding domain and caused loss of the dimerization domain, while 64.3% of them only disrupted the dimerization domain (Fig. 1A, Additional file 4: Table S1). As indicated, *IKZF1* mutation was recurrent in AML, but its role in AML pathogenesis needed further investigations.

**Fig. 1:**
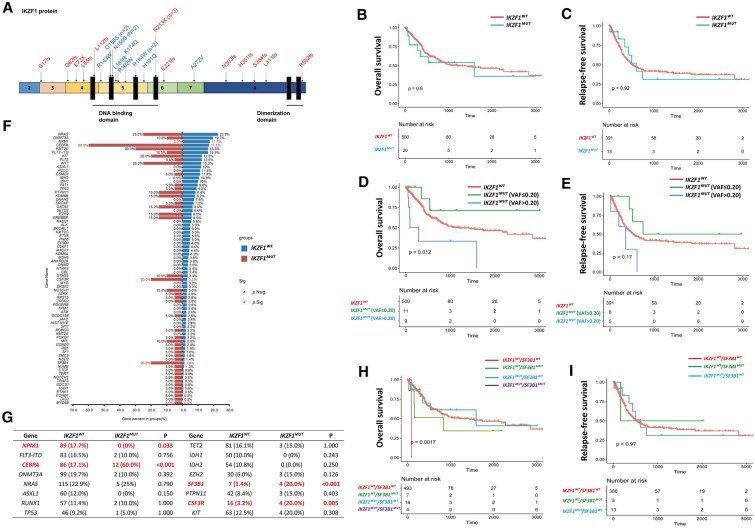
*IKZF1* mutation in AML. (**A**) The distribution of *IKZF1* mutations, which were identified in our cohort, on the protein. The nonsense or frameshift mutation was marked as red, while the missense mutation was marked as blue. (**B, C**) The OS (**B**) and RFS (**C**) of *IKZF1*^*WT*^ and *IKZF1*^*MUT*^ groups in our AML cohort. (**D**, **E**) The influence of *IKZF1* mutation burden on the prognosis of AML was studied, and the OS (**D**) as well as RFS (**E**) of *IKZF1*^*WT*^, *IKZF1*^*MUT*^ with VAF > 0.20, and *IKZF1*^*MUT*^ with VAF ≤ 0.20 groups are shown. (**F**) The difference of additional mutations distribution in *IKZF1*^*WT*^ and *IKZF1*^*MUT*^ groups, and the percentage of each gene mutation is exhibited. (**G**) The distribution of frequent AML-associated gene mutations in *IKZF1*^*WT*^ and *IKZF1*^*MUT*^ groups, and the count as well as percentage of each gene mutation are shown. (**H**, **I**) The prognostic role of combined *IKZF1* and *SF3B1* mutations on AML was investigated, and the OS (**H**) as well as RFS (**I**) of AML with different *IKZF1* or *SF3B1* mutated status are exhibited

To investigate the features of *IKZF1*^*MUT*^ AML, we compared the baseline characteristics of the *IKZF1*^*MUT*^ and *IKZF1*^*WT*^ groups, and the only difference was found in median age. ELN 2017 prognostic stratification predicted the clinical outcome of AML patients well [2]. Compared to the *IKZF1*^*WT*^ group, the *IKZF1*^*MUT*^ group showed a higher frequency of patients in the ELN-intermediate-risk group and a lower frequency in the ELN-low-risk and ELN-high-risk groups, but the CR rate in the *IKZF1*^*MUT*^ group was significantly lower than that in the *IKZF1*^*WT*^ group under our treatment strategy (Table 1). More interestingly, *IKZF1*^*MUT*^ patients showed similar OS and RFS with *IKZF1*^*WT*^ patients (Fig. 1B, C). Though *IKZF1* mutation conferred one disadvantaged therapeutic response for AML patients, overall, it finally did not influence their survival time.

**Table 1:** Baseline characteristics of our AML cohort

Characteristics	*IKZF1*^*WT*^ group	*IKZF1*^*MUT*^ group	P
N, % of total	502, 96.2%	20, 3.8%	
Age (years)	50.0 (11.0–82.0)	42.5 (15.0–66.0)	0.032
Gender			
Male (N)	220 (43.8%)	10 (50%)	0.585
Female (N)	282 (56.2%)	10 (50%)	
Peripheral blood			
White blood cells (10^9^/L)	11.30 (0.40–484.80)	17.72 (1.67–120.00)	0.533
Hemoglobin (g/L)	83.00 (20.00–204.00)	92.00 (57.00–148.00)	0.130
Platelets (10^9^/L)	51.00 (2.00–565.00)	63.00 (7.00–917.00)	0.633
Bone marrow blasts (%)	59.5 (11.5–98.0)	59.0 (20.0–96.0)	0.559
Diagnosis (N)			
De novo AML	481 (95.8%)	19 (95.0%)	0.584
Secondary/therapy-related AML	21 (4.2%)	1 (5.0%)	
French-American-British (N)			
M0	21 (4.2%)	2 (10.0%)	0.895
M1	27 (5.4%)	1 (5.0%)	
M2	170 (33.9%)	7 (35.0%)	
M4	101 (20.1%)	5 (25.0%)	
M5	161 (32.1%)	5 (25.0%)	
M6	11 (2.2%)	0 (0%)	
M7	1 (0.2%)	0 (0%)	
Undefine	10 (2.0%)	0 (0%)	
Cytogenetics (N)			
Normal karyotype	247 (49.2%)	10 (50.0%)	0.944
Complex karyotype	44 (8.8%)	2 (10.0%)	0.693
Monosomal karyotype	16 (3.2%)	0 (0%)	1.000
-5/5q-/monosomy 5	21 (4.2%)	0 (0%)	1.000
-7/monosomy 7	17 (3.3%)	0 (0%)	1.000
-17/17p abnormalities	11 (2.2%)	0 (0%)	1.000
Chromosome 3 abnormalities	16 (3.2%)	2 (10.0%)	0.148
Gene fusions (N)			
*RUNX1::RUNX1T1*	65 (12.9%)	1 (5.0%)	0.494
*CBFB::MYH11*	36 (7.2%)	0 (0%)	0.386
*BCR::ABL1*	9 (1.8%)	0 (0%)	1.000
*KMT2A* rearrangements	19 (3.8%)	0 (0%)	1.000
European Leukemia Net 2017 (N)			
Low	151 (30.1%)	2 (10.0%)	0.004
Intermediate	212 (42.2%)	16 (80.0%)	
High	139 (27.7%)	2 (10.0%)	
Complete remission (N)	394 (78.5%)	13 (65.0%)	0.032
No complete remission (N)	73 (21.5%)	7 (35.0%)	

To interpret the contrast phenomena and define the prognostic role of *IKZF1* mutation more clearly, we analyzed the influence of its VAF, mutational type, and mutational count on the duration of survival. We performed maximally selective log-rank statistics in OS based on VAF and found that *IKZF1*^*MUT*^ patients with a high *IKZF1* VAF burden (VAF > 0.20) showed significantly poorer OS than those with low VAF or *IKZF1*^*WT*^, but the RFS did show any statistically significant difference (Fig. 1D, E, Additional file 5: Table S2). We found that neither the type nor the number of mutations influenced OS or RFS in *IKZF1*^*MUT*^ patients (Additional file 1: Fig S1C–F). In this way, a high burden of *IKZF1* mutation might predict poor prognosis in AML.

To exclude the impact of additional factors on OS, we performed univariate and multivariate analyses that included baseline characteristics and genetic alterations. In univariate analysis, we identified 20 factors that had a significant influence on OS in our AML cohort, including *IKZF1* mutations with high VAF. In multivariate analysis, we strongly indicated that *IKZF1* mutation with high VAF was one independent risk factor for the death of AML (HR, 6.101; 95% CI 2.278–16.335; P = 0.0003) (Additional file 6: Table S3).

We also analyzed the relationships among *IKZF1* mutation and other gene mutations. *IKZF1* mutation exhibited concurrences with *CEBPA*, *SF3B1*, and *CSF3R* mutations, but it was mutually exclusive with *NPM1* mutation (Fig. 1F, G). We also performed subgroup survival analysis. The prognostic role of *CEBPA*^*bZIP−inf*^ [3–5], *SF3B1*, and *CSF3R* mutations was revealed in our cohort (Additional file 2: Fig S2). *IKZF1* mutation did not influence OS or RFS in *CSF3R*^*WT*^ and *CSF3R*^*MUT*^ (Additional file 3: Fig S3A, B, Additional file 7: Table S4). In *IKZF1*^*MUT*^ patients, *CEBPA*^*bZIP−inf*^ mutation (83.3%) was more common than non-*CEBPA*^*bZIP−inf*^ mutation (16.7%). *IKZF1* mutation conferred a relatively low CR in the *CEBPA*^*WT*^*/*non-*CEBPA*^*bZIP−inf−MUT*^ group, but not in the *CEBPA*^*bZIP−inf−MUT*^ group (Additional file 8: Table S5), and it influenced OS and RFS in the *CEBPA*^*WT*^*/*non-*CEBPA*^*bZIP−inf−MUT*^ group but not in the *CEBPA*^*bZIP−inf−MUT*^ group (Additional file 3: Fig S3C, D). *IKZF1*^*WT*^/*SF3B1*^*MUT*^ AML patients exhibited a CR rate of 50%, and the therapeutic response was even worse in *IKZF1*^*MUT*^/*SF3B1*^*MUT*^ AML. None of these patients achieved CR at any point during the regimen (Additional file 9: Table S6). *IKZF1* mutation combined with *SF3B1* mutation conferred extremely poor OS on AML, but the RFS of *IKZF1*^*MUT*^/*SF3B1*^*MUT*^ AML patients was unavailable because no patient reached CR (Fig. 1H, I).

Compared with foreign cohorts (OHSU [6], 1.35%; TCGA [7], 0.5%; TARGET [8], 4.21%), the frequency of *IKZF1* mutation was relatively high (3.83%). This may be because patients were of different races or it may be because of differences in sequencing depth. *IKZF1* deletion, caused by -7/monosomy 7, was detected in 3.20% of our patients. Unlike in ALL [9], *IKZF1* mutation and deletion were equally dominant in AML [10]. Missense mutation accounted for nearly half of *IKZF1* mutations, and it almost affected the DNA-binding domain in AML, while its DNA-binding domain and dimerization domain involvement was relatively balanced in ALL [9]. *IKZF1* aberration conferred poor prognosis in ALL [11], but only a high burden of *IKZF1* mutation predicted poor OS in AML because *IKZF1* mutation with VAF < 10% accounted for 35% of all *IKZF1*^*MUT*^ patients, and *IKZF1* mutation contributed less to disease than other mutations did in this group of patients. *CEBPA* mutation was the most common co-mutation that occurred alongside *IKZF1* mutation in AML [1, 12].

## Supplementary Information

**Additional file 1: Fig S1.** The mutational landscape of our AML cohort. (A) Frequent mutations with more than 10 counts in our cohort were showed. (B) The relationship between mutations was analyzed, concurrent and mutually-exclusive mutations were indicated. (C) The concurrent or mutually-exclusive mutations for common rearrangements in AML were exhibited.

**Additional file 2: Fig S2.** The prognostic role of CSF3R, CEBPAbZIP-inf, or SF3B1 mutation in AML. (A-B) The prognostic role of CSF3R mutation in AML, and OS (A) as well as PFS (B) were showed. (C-D) The OS (C) and PFS (D) of patients with CEBPAWT plus non-CEBPAbZIP-inf mutation or CEBPAbZIP-inf mutation in AML. (E-F) The OS (E) and RFS (F) of SF3B1WT and SF3B1MUT groups in our AML cohort.

**Additional file 3: Fig S3.** The prognostic role of IKZF1 mutation in the specific genetic AML subtype. (A-B) The influence of IKZF1 mutation on the OS (A) and PFS (B) of CSF3R-mutated AML. (C-D) The influence of IKZF1 mutation on the prognosis of the CEBPA-mutated AML was studied, and the OS (A) as well as RFS (B) of CEBPAWT plus non-CEBPAbZIP-inf-MUT and CEBPAbZIP-inf-MUT groups with or without IKZF1 mutation were showed.

**Additional file 4: Table S1.** IKZF1-mutated AML patients in our cohort.

**Additional file 5: Table S2.** The CR rate of AML with different burdens of IKZF1 mutation.

**Additional file 6: Table S3.** Univariate and multivariate analysis for overall survival duration.

**Additional file 7: Table S4.** The influence of IKZF1 mutation on AML with different CSF3R-mutated status.

**Additional file 8: Table S5.** The influence of IKZF1 mutation on AML with different CEBPA-mutated status.

**Additional file 9: Table S6.** The influence of IKZF1 mutation on AML with different SF3B1-mutated status.

## Data Availability

The datasets used and/or analyzed during the current study are available from the corresponding author on reasonable request.

## Abbreviations

ALL: Acute lymphoblastic leukemia
AML: Acute myeloid leukemia
CI: Confidence interval
CR: Complete remission
ELN: European Leukemia Net
HR: Hazard ratio
IKZF1^MUT^: IKZF1-mutated
IKZF1^WT^: IKZF1-wild type
*CEBPA*^*bZIP*^^*−*^^*inf*^^*−*^^*MUT*^: In-frame bZIP *CEBPA*-mutated
OS: Overall survival
RFS: Relapse-free survival
VAF: Variant allele frequency
