# Supplementary figures and images for: The clinical impact of IKZF1 mutation in acute myeloid leukemia
Source: Exp Hematol Oncol. 2023 Mar 30;12:33. doi: 10.1186/s40164-023-00398-y (PMC10061890; doi:10.1186/s40164-023-00398-y)

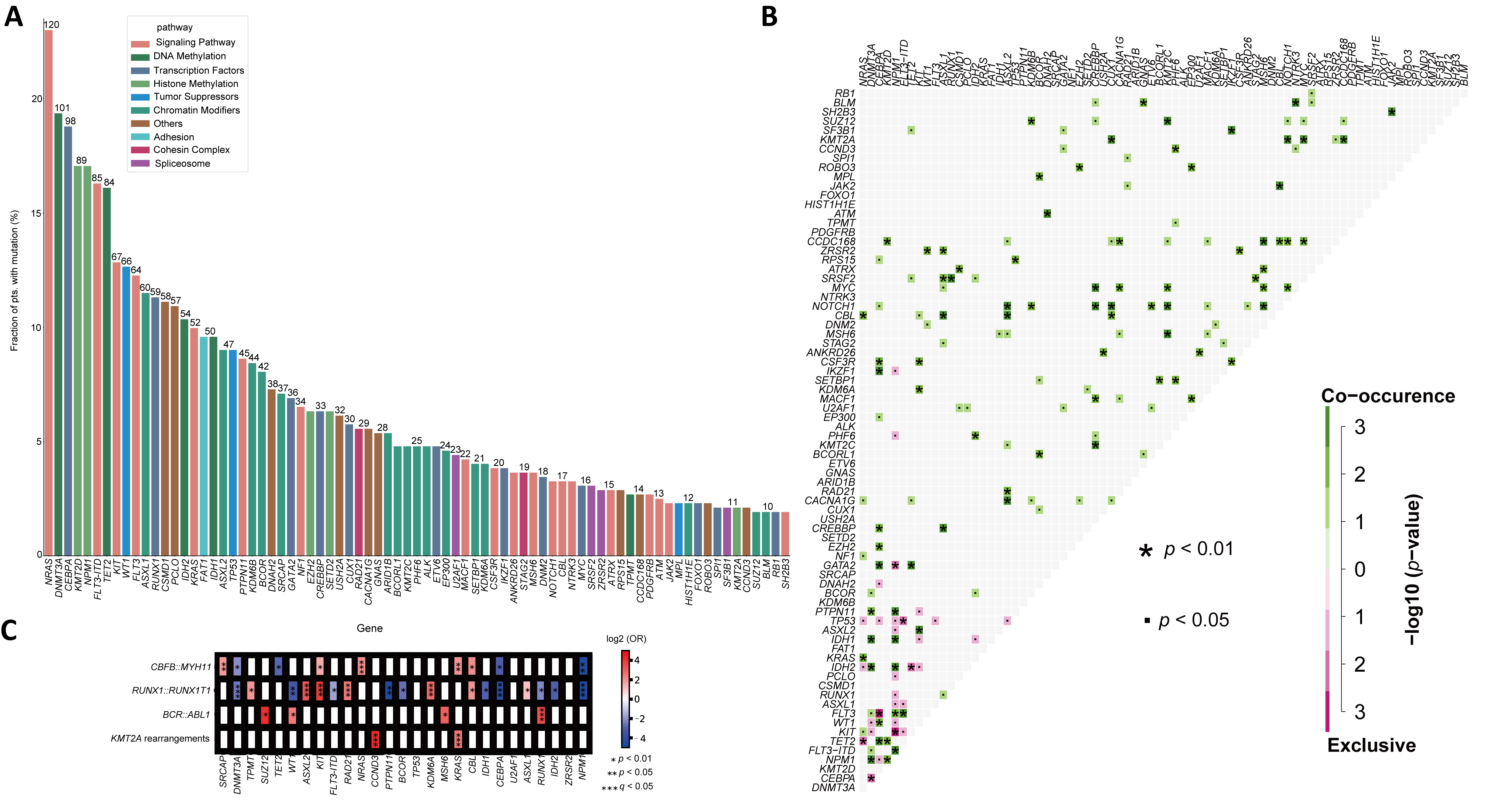

Supplement: Supplementary file 1 — Additional file 1: Fig S1. The mutational landscape of our AML cohort. (A) Frequent mutations with more than 10 counts in our cohort were showed. (B) The relationship between mutations was analyzed, concurrent and mutually-exclusive mutations were indicated. (C) The concurrent or mutually-exclusive mutations for common rearrangements in AML were exhibited. [file 40164_2023_398_MOESM1_ESM.tif]

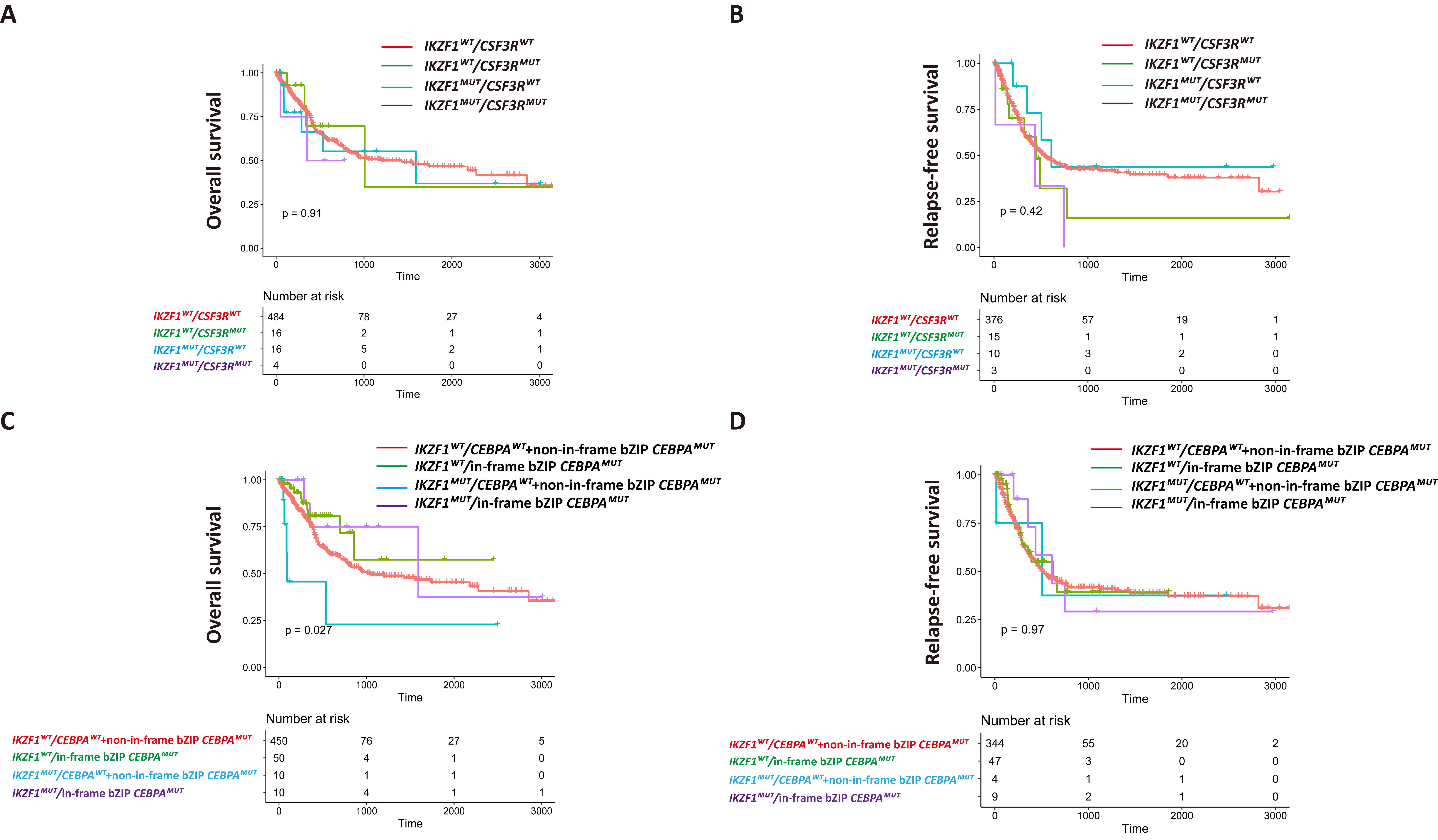

Supplement: Supplementary file 3 — Additional file 3: Fig S3. The prognostic role of IKZF1 mutation in the specific genetic AML subtype. (A-B) The influence of IKZF1 mutation on the OS (A) and PFS (B) of CSF3R-mutated AML. (C-D) The influence of IKZF1 mutation on the prognosis of the CEBPA-mutated AML was studied, and the OS (A) as well as RFS (B) of CEBPAWT plus non-CEBPAbZIP-inf-MUT and CEBPAbZIP-inf-MUT groups with or without IKZF1 mutation were showed. [file 40164_2023_398_MOESM3_ESM.tif]
