# Supplementary material for: The clinical impact of IKZF1 mutation in acute myeloid leukemia
Source: Exp Hematol Oncol. 2023 Mar 30;12:33. doi: 10.1186/s40164-023-00398-y (PMC10061890; doi:10.1186/s40164-023-00398-y)
Supplement: Supplementary file 4 — Additional file 4: Table S1. IKZF1-mutated AML patients in our cohort. [file 40164_2023_398_MOESM4_ESM.docx]

**Table S1. *IKZF1*-mutated AML patients in our cohort**

| **UPN** | **Age/Sex** | **Diagnosis** | **Karyotype** | ***IKZF1* mutation** | | | **Other genetic mutation** |
| --- | --- | --- | --- | --- | --- | --- | --- |
|  |  |  |  | **MutFreq** | **cHGVS** | **pHGVS** |  |
| 1 | 60/M | *de novo* AML | 46,XY | 0.01 | c.1233delC | p.L411fs | *MPL, CEBPA, CCDC168* |
|  |  |  |  | 0.20 | c.1054dupA | p.H351fs |  |
| 2 | 52/F | *de novo* AML | 46,XX,t(8;21) | 0.02 | 550C>T | p.R184W | *NRAS, KRAS, ASXL2* |
| 3 | 45/M | *de novo* AML | 46,XY | 0.45 | c.49_50insGC | p.S17fs | *NOTCH1, DNMT3A, CSF3R* |
| 4 | 30/F | *de novo* AML | 46,XX | 0.03 | c.1505_1511del | p.R502fs | *CEBPA* |
|  |  |  |  | 0.02 | 637C>T | p.R213X |  |
| 5 | 23/M | *de novo* AML | 46,XY,9q- | 0.44 | c.663delG | p.E221fs | *SRCAP, FLT3-ITD, CEBPA* |
| 6 | 52/F | *de novo* AML | 45-47,XX,der(1),der(5),7p-,+8,r(12),-13,13q-,-15,18q-,-22,+20q-[cp18]/46,XX[2] | 0.23 | 476A>G | p.N159S | *TP53* |
| 7 | 55/F | *de novo* AML | 46,XX | 0.40 | c.909_910del | p.N303fs | *NRAS, KIT, CEBPA* |
| 8 | 34/F | *de novo* AML | NA | 0.04 | c.253_260del | p.L85fs | *KRAS, KIT, FLT3-ITD, CSF3R, CEBPA* |
| 9 | 40/M | *de novo* AML | 46,XY,t(11;19) | 0.46 | 573C>A | p.H191Q | *WT1, TET2, NRAS, KIT, FAT1, CEBPA* |
| 10 | 23/M | *de novo* AML | 46,XY | 0.06 | 815C>T | p.A272V | *STAG2, NRAS, NF1* |
| 11 | 28/F | *de novo* AML | 45,X,-X | 0.11 | 484C>T | p.R162W | *NRAS, KIT, EZH2, CSF3R, CEBPA* |
| 12 | 52/F | *de novo* AML | 46,XX[20] | 0.43 | 476A>G | p.N159S | *SF3B1, PTPN11, ETNK1* |
| 13 | 61/M | *de novo* AML | 47,XY,+3(q21)[20] | 0.38 | c.214G>T | p.E72X | *SF3B1, PTPN11, FLT3, BCOR* |
|  |  |  |  | 0.42 | c.1150delT | p.S384fs |  |
| 14 | 45/M | *de novo* AML | 46,XY,der(3)(q27)[10] | 0.45 | c.184_185insAA | p.Q62fs | *SF3B1, PTPN11* |
|  |  |  |  | 0.47 | 550C>T | p.R184W |  |
| 15 | 24/M | *de novo* AML | 46,XY,del(8)(q22){5}/46,XY{5} | 0.02 | 427C>T | p.R143W | *WT1, CEBPA* |
|  |  |  |  | 0.03 | 637C>T | p.R213X |  |
| 16 | 34/F | *de novo* AML | 46,XX[20] | 0.20 | c.336delinsGCCCG | p.L112fs | *WT1, CTCF, CSF3R, CEBPA* |
| 17 | 50/M | s/t-AML | 46,XY[20] | 0.04 | c.637C>T | p.R213X | *WT1, TET2, SF3B1, GATA2, DNMT3A* |
| 18 | 23/M | *de novo* AML | 46,XY[20] | 0.49 | 472G>A | p.G158S | *RUNX1, GATA2, CEBPA* |
| 19 | 15/F | *de novo* AML | 46,XX,-2,-6,-11,del(13)(q13q22), +3mar[6]/46,XX[4] | 0.03 | c.520A>C | p.K174Q | *CCND3* |
| 20 | 66/F | *de novo* AML | 46,XX[20] | 0.01 | c.472G>A | p.G158S | / |
|  |  |  |  | 0.14 | c.482T>G | p.L161R |  |

**M**, male; **F**, female; **AML**, acute myeloid leukemia; **MLL**, mix lineage leukemia; **s/t-AML**, secondary/therapy-related AML; **NA**, not available.
