# Supplementary material for: The clinical impact of IKZF1 mutation in acute myeloid leukemia
Source: Exp Hematol Oncol. 2023 Mar 30;12:33. doi: 10.1186/s40164-023-00398-y (PMC10061890; doi:10.1186/s40164-023-00398-y)
Supplement: Supplementary file 5 — Additional file 5: Table S2. The CR rate of AML with different burdens of IKZF1 mutation. [file 40164_2023_398_MOESM5_ESM.docx]

**Table S2. The CR rate of AML with different burdens of *IKZF1* mutation**

| **Characteristic** | **CR** | **Non-CR** | **P** |
| --- | --- | --- | --- |
| Maximum variant allele frequency |  |  |  |
| ≤ 0.20 | 8 (72.7%) | 3 (27.3%) | 0.642 |
| > 0.20 | 5 (55.6%) | 4 (44.4%) |  |
