# Supplementary material for: The clinical impact of IKZF1 mutation in acute myeloid leukemia
Source: Exp Hematol Oncol. 2023 Mar 30;12:33. doi: 10.1186/s40164-023-00398-y (PMC10061890; doi:10.1186/s40164-023-00398-y)
Supplement: Supplementary file 6 — Additional file 6: Table S3. Univariate and multivariate analysis for overall survival duration. [file 40164_2023_398_MOESM6_ESM.docx]

**Table S3. Univariate and multivariate analysis for overall survival duration**

| Variable | Univariate | | Multivariate | |
| --- | --- | --- | --- | --- |
|  | HR | P | HR | P |
| ELN adverse risk (poor vs. intermediate vs. good) | 1.720 | 0.00000001 | - | - |
| *TP53* mutation | 2.654 | 0.000004 | 2.182 | 0.003 |
| *CBF*-AML | 0.356 | 0.000008 | 0.459 | 0.005 |
| Advanced age (≥60 years old) | 1.995 | 0.00003 | 1.716 | 0.006 |
| *U2AF1* mutation | 2.646 | 0.001 | 3.156 | 0.001 |
| Aberrant karyotype | 1.913 | 0.001 | 2.35 | 0.0002 |
| *DNMT3A* mutation | 1.796 | 0.001 | 1.674 | 0.014 |
| High WBC count (≥11.8 x 10^9^/L) | 1.646 | 0.002 | 2.07 | 0.00007 |
| BMT | 0.560 | 0.002 | 0.557 | 0.007 |
| *IKZF1* mutation with high VAF (> 0.20) | 2.943 | 0.006 | 6.101 | 0.0003 |
| *ASXL2* mutation | 0.459 | 0.010 | 0.562 | 0.114 |
| *KMT2A* rearrangements | 2.180 | 0.014 | 1.791 | 0.103 |
| *BCOR* mutation | 1.722 | 0.027 | 0.978 | 0.935 |
| *KRAS* mutation | 1.586 | 0.042 | 1.521 | 0.113 |
| *SF3B1* mutation | 2.266 | 0.043 | 1.941 | 0.164 |
| *SRCAP* mutation | 0.489 | 0.043 | 0.468 | 0.050 |
| *CEBPA^bZIP-inf^* mutation | 0.574 | 0.061 | 0.459 | 0.045 |
| *DNM2* mutation | 0.354 | 0.062 | 0.633 | 0.444 |
| *BCORL1* mutation | 1.800 | 0.067 | 1.476 | 0.290 |
| *FLT3-ITD* mutation | 1.396 | 0.075 | 1.153 | 0.520 |

**ELN**, European Leukemia Net; **CBF**, core-binding factor; **WBC**, white blood cells; **BMT**, bone marrow transplantation.
