# Supplementary material for: The clinical impact of IKZF1 mutation in acute myeloid leukemia
Source: Exp Hematol Oncol. 2023 Mar 30;12:33. doi: 10.1186/s40164-023-00398-y (PMC10061890; doi:10.1186/s40164-023-00398-y)
Supplement: Supplementary file 7 — Additional file 7: Table S4. The influence of IKZF1 mutation on AML with different CSF3R-mutated status. [file 40164_2023_398_MOESM7_ESM.docx]

**Table S4. The influence of *IKZF1* mutation on AML with different *CSF3R*-mutated status.**

| **Characteristic** | **CR** | **Non-CR** | **P** |
| --- | --- | --- | --- |
| *IKZF1^WT^, CSF3R^WT^* | 379 (83.8%) | 73 (16.2%) | 0.634 |
| *IKZF1^WT^, CSF3R^MUT^* | 15 (100%) | 0 (0%) | 0.147 |
| *IKZF1^MUT^, CSF3R^WT^* | 10 (62.5%) | 6 (37.5%) | 0.033 |
| *IKZF1^MUT^, CSF3R^MUT^* | 3 (75.0%) | 1 (25.0%) | 0.513 |
