# Supplementary material for: The clinical impact of IKZF1 mutation in acute myeloid leukemia
Source: Exp Hematol Oncol. 2023 Mar 30;12:33. doi: 10.1186/s40164-023-00398-y (PMC10061890; doi:10.1186/s40164-023-00398-y)
Supplement: Supplementary file 8 — Additional file 8: Table S5. The influence of IKZF1 mutation on AML with different CEBPA-mutated status. [file 40164_2023_398_MOESM8_ESM.docx]

**Table S5. The influence of *IKZF1* mutation on AML with different *CEBPA*-mutated status**

| **Characteristic** | **CR** | **Non-CR** | **P** |
| --- | --- | --- | --- |
| *IKZF1^WT^, CEBPA^WT^/*non-*CEBPA^bZIP-inf-MUT^* | 347 (83.2%) | 70 (16.8%) | 0.601 |
| *IKZF1^WT^, CEBPA^bZIP-inf-MUT^* | 47 (94.0%) | 3 (6.0%) | 0.036 |
| *IKZF1^MUT^, CEBPA^WT^/*non-*CEBPA^bZIP-inf-MUT^* | 4 (40.0%) | 6 (60.0%) | 0.002 |
| *IKZF1^MUT^, CEBPA^bZIP-inf-MUT^* | 9 (90.0%) | 1 (10.0%) | 1.000 |
