# Supplementary material for: The clinical impact of IKZF1 mutation in acute myeloid leukemia
Source: Exp Hematol Oncol. 2023 Mar 30;12:33. doi: 10.1186/s40164-023-00398-y (PMC10061890; doi:10.1186/s40164-023-00398-y)
Supplement: Supplementary file 9 — Additional file 9: Table S6. The influence of IKZF1 mutation on AML with different SF3B1-mutated status. [file 40164_2023_398_MOESM9_ESM.docx]

**Table S6. The influence of *IKZF1* mutation on AML with different *SF3B1*-mutated status.**

| **Characteristic** | **CR** | **Non-CR** | **P** |
| --- | --- | --- | --- |
| *IKZF1^WT^, SF3B1^WT^* | 391 (85.0%) | 69 (15.0%) | 0.004 |
| *IKZF1^WT^, SF3B1^MUT^* | 3 (42.9%) | 4 (57.1%) | 0.016 |
| *IKZF1^MUT^, SF3B1^WT^* | 13 (81.3%) | 3 (18.7%) | 0.735 |
| *IKZF1^MUT^, SF3B1^MUT^* | 0 (0%) | 4 (100%) | 0.001 |
